# *Alloiococcus otitidis*—Cause of Nonspecific Acute Sinusitis: First Case Report and Review of Literature

**DOI:** 10.3390/microorganisms10061182

**Published:** 2022-06-09

**Authors:** Tanja Grubić Kezele, Maja Abram, Marina Bubonja-Šonje

**Affiliations:** 1 Department of Clinical Microbiology, Clinical Hospital Center Rijeka, Krešimirova 42, 51000 Rijeka, Croatia; maja.abram@medri.uniri.hr (M.A.); marina.bubonja@uniri.hr (M.B.-Š.); 2 Department of Physiology, Immunology and Pathophysiology, Faculty of Medicine, University of Rijeka, Braće Branchetta 20, 51000 Rijeka, Croatia; 3 Department of Microbiology and Parasitology, Faculty of Medicine, University of Rijeka, Braće Branchetta 20, 51000 Rijeka, Croatia

**Keywords:** acute sinusitis, *Alloiococcus otitidis*, nasopharyngeal microbiota

## Abstract

Although most sinus infections are viral, potential bacterial pathogens such as *Streptococcus pneumoniae*, *Haemophilus influenza* and *Moraxella catarrhalis* can migrate during a viral respiratory infection from the nasopharynx into the sinus cavity causing sinusitis. *Alloiococcus otitidis* is a commensal of the external auditory canal and is considered one of the potential middle ear pathogens. Unlike most otopathogens, *A. otitidis* is rarely found in the nasopharynx of healthy individuals. This difficult-to-culture organism has not previously been described as a causative agent of sinusitis. Here we describe one case of acute sinusitis due to *A. otitidis* and review previous knowledge of this controversial organism based on recent literature.

## 1. Introduction

The nasopharyngeal microbiota is very diverse and varies from person to person. The upper respiratory tract, including the nasopharynx, serves as a reservoir for pathogens that can cause respiratory infections, including sinusitis [1]. Although most sinus infections in children are viral in nature, potential bacterial pathogens can migrate from the nasopharynx into the sinuses during a viral respiratory infection and cause sinusitis [2]. The most common bacteria isolated in pediatric patients with acute sinusitis are *Streptococcus pneumoniae*, *Haemophilus influenzae*, *Moraxella catarrhalis*, and *Streptococcus pyogenes* [3].

*Alloiococcus otitidis* was first isolated from the middle ear fluid (MEF) of children with acute otitis media (OM) by Faden and Dray in 1989 [4]. Since then, it has been frequently detected in MEF specimens and suspected as a cause of OM. However, more than three decades later, its possible role in this disease remains controversial. There is still insufficient evidence to clarify whether *A. otitidis* is a commensal or a true pathogen, or perhaps indirectly contributes to the pathogenesis of OM by supporting important pathogens in some way [5,6].

Acute bacterial sinusitis and OM have identical pathogenesis: an antecedent viral infection and nasopharyngeal colonization by bacterial pathogens. Although frequently detected in MEFs in recent decades, *A. otitidis* has very rarely, if ever, been detected in nasopharyngeal cultures from healthy individuals or patients with respiratory infections [6,7]. However, with recent advances in culture-independent techniques such as 16S rRNA gene sequencing, *Alloiococcus* has been detected as a common genus in the microbiota of the healthy nasopharynx [8]. The same study also revealed that the microbiota of the healthy nasopharynx is most similar to the microbiota of the external auditory canal [8]. However, *Alloiococcus* is most closely related to *Dolosigranulum* by 16S rRNA sequence homology [6]. *Dolosigranulum* is a nasopharyngeal commensal of relevance to OM, and its similarity leads to misclassification in 16S rRNA gene studies using older taxonomic databases [6]. This must be taken into account when using 16S rRNA analysis.

To our knowledge, this difficult-to-culture organism has not been described in the literature as a causative agent of acute sinusitis in children or adults. We describe here a case of acute sinusitis attributable to *A. otitidis* and review previous knowledge of this organism based on the current literature.

## 2. Case Report

A 9-year-old boy with no significant past medical history (allergic rhinitis only) was brought to the Department of Otolaryngology-Head and Neck Surgery in February 2020 complaining of a headache in the frontal area above the frontal sinus that had been present for 10 days. The patient had previously received intranasal Maxitrol^®^ drops containing neomycin, polymyxin B, and dexamethasone (Novartis Pharmaceuticals, Basel, Switzerland) for two weeks, after which he experienced relief of symptoms. For his allergy, he received Nisita^®^ (physiological saline nasal spray; Engelhard Arzneimittel GmbH & Co. KG., Niederdorfelden, Germany), Auerius^®^ (desloratadine; Schering-Plough Central East Ag, Zürich, Switzerland), and Melarth^®^ (montelukast sodium; Pliva d.o.o., Zagreb, Croatia). We assume these medications minimized other sinusitis symptoms such as mucopurulent discharge and more severe inflammation with obstruction. On examination, the boy was afebrile, otoscopically normal, rhinoscopically with edematous and inflamed mucosa without pathologic mucopurulent discharge, and oropharyngoscopically with the present catarrh and visible tonsils occupying 50% of the pharynx. Sinus aspiration was performed according to the Proetz method and the sinus aspirate was sent to the microbiology laboratory for culture. The patient was instructed to take Maxitrol^®^ drops (3 × 2 drops per day for 5 days) until the results of the microbiological analysis were available, and to take the amoxicillin prescribed by the general practitioner in case of fever.

The aspirate was cultured on 5% sheep blood agar, chocolate agar and Columbia agar (BD Difco™, Le Pont-de-Claix, France) at 35 °C for 48 h aerobically with 5% CO_2_ and anaerobically. Small, moist, weakly catalase-positive, alpha-hemolytic colonies grew in pure culture after 48 h aerobically. It should be noted that colonies were too small to see without a magnifying glass. Gram staining revealed small Gram-positive cocci. *A. otitidis* was identified using the VITEK 2 microbial identification system (bioMérieux, Marcy l’Etoile, France). Antimicrobial susceptibility testing was performed by disk diffusion on Müller -Hinton agar (Oxoid, Basingstoke, UK) spiked with 5% sheep blood. Since breakpoints are not defined for *A. otitidis*, the EUCAST standard for *S. pneumoniae* was used to interpret the results [9]. The isolate showed sensitivity to ampicillin, amoxicillin, cefuroxime, ceftriaxone, sulfamethoxazole-trimethoprim, vancomycin, teicoplanin, and tetracycline, and resistance to erythromycin, azithromycin, clarithromycin, and clindamycin.

After completion of microbiological analysis and antimicrobial susceptibility testing, the patient underwent endoscopic examination, which revealed hypertrophic and livid turbinates, enlarged adenoids, and increased nonpurulent nasal discharge.

The patient was administered amoxicillin 1000 mg orally twice daily for 10 days. At follow-up two weeks later, the patient recovered (subjective improvement with otoscopic and oropharyngoscopic normal findings and rhinoscopic pre-existing signs of allergic and vasomotor rhinitis) with no recurrence at follow-up two months later. No additional microbiological control examinations were performed.

## 3. Discussion and Literature Review

A thorough literature search was performed in the MEDLINE database for case reports and reviews published in English-language peer-reviewed journals using MeSH terms. A search for “*Aleiococcus otitidis*” as a keyword yielded 66 articles, whereas the keywords “*Aleiococcus otitidis*” and “otitis media” yielded 55 articles. In addition, Table 1 summarizes all studies in which *Alloiococcus otitidis* was detected in nasopharyngeal specimens. Searching for the keywords “*Aleiococcus otitidis*” and “sinusitis” yielded only one article, published in 2003 by Kalcioglu et al. [5]. The authors reported the detection of *A. otitidis* by polymerase chain reaction (PCR) in two specimens with chronic sinusitis and suggested that this organism could cause chronic sinusitis. However, no case of acute sinusitis caused by *A. otitidis* has been reported to date.

*A. otitidis* is the only species of the genus *Alloiococcus.* This difficult-to-culture Gram-positive coccus is an aerobic, slow-growing bacterium that was originally isolated from MEF by microbiological culture and considered to be the cause of OM [10]. In the last two decades, it has been frequently detected in the middle ear of children with OM, along with other common otitis-associated bacteria or alone [7,10,11,12,13]. Since its discovery, it has been highly controversial as to whether *A. otitidis* is truly a pathogen or merely a commensal of the external auditory canal [6]. This is mainly because the organism could be found in the external auditory canal (EAC) of apparently healthy individuals [14]. In a recent study, Sjovall et al., reported that EAC samples with a high relative abundance of *Staphylococcus* typically had a low abundance of *Alloiococcus* and vice versa. The authors hypothesized that these two genera should be considered competing members of the microbial community.

Moreover, *A. otitidis* was rarely detected in the nasopharynx of healthy individuals, where the major otopathogens (*S. pneumoniae*, *H. influenzae*, and *M. catarrhalis*) mainly colonize. Nasopharyngeal colonization is a necessary first step in the pathogenesis of acute OM. However, the reason for the rarity of *A. otitidis* in clinical specimens may simply be the failure to culture this fastidious organism. The organism is difficult to isolate using conventional culture methods, and the use of culture-independent methods has greatly improved the detection rate of this bacterium in respiratory specimens. Recently, the high prevalence of the genus *Alloiococcus* in the nasopharyngeal microbiota of otitis-prone children compared with healthy controls was demonstrated by 16S rRNA analysis [13].

In the previously cited article by Kalcioglu et al., the detection of *A. otitidis* DNA in sinus aspirates from two patients with chronic sinusitis was confirmed [5]. Also, in another study using the PCR method, the presence of *A. otitidis* in effusions from OM correlated with persistent inflammation of the tympanic mucosa [10]. This may suggest that the bacterium is less important in acute infections and more associated with chronicity [5].

However, it cannot be ruled out that *A. otitidis* is present in the acute phase of these two diseases but is overlooked by conventional culture-based methods because of its slow growth (see Table 1). Although its pathogenicity has not yet been proven, Chan et al. have shown that *A. otitidis* can form a biofilm through bacterial interaction with *H. influenzae*, thus indirectly contributing to pathogenicity [15]. *A. otitidis* forms a biofilm with both *H. influenzae* and other species. In addition, *A. otitidis* in a polymicrobial biofilm can promote the growth and survival of *H. influenzae* by increasing biofilm production under unfavorable growth conditions and altering antimicrobial resistance. This study of bacterial interference by biofilm showed an inverse ratio of the relative abundance of *A. otitidis* and *H. influenzae*, suggesting a possible bacterial interaction in patients with sinusitis or OM. Accordingly, chronic sinusitis can be considered as a disease caused by biofilm [15]. Namely, the bacteria may cause subacute inflammation during biofilm formation, thus promoting sinusitis. Moreover, biofilm gives bacteria a survival advantage and makes them resistant to antibiotics, phagocytosis, and humoral immunity [15,16,17,18]. Thus, we can assume that one of the most important hypotheses for the etiology of chronic rhinosinusitis is bacterial biofilm formation, which includes *A. otitidis* among other bacteria.

In addition, there are many studies describing the ability of *A. otitidis* to stimulate an immune response. These results indicate its pathogenic potential and preliminary evidence that the immune system responds to it. Thus, *A. otitidis* was able to stimulate the production of IL-8 and IL-12 to a similar extent as *M. catarrhalis*, *S. pneumoniae,* and *H. influenzae* (important otopathogens) [19,20]. The elicitation of an inflammatory response suggests that *A. otitidis* may be a pathogen. Moreover, MEFs positive for *A. otitidis* contained similar levels of proinflammatory cytokines as MEFs positive for *S. pneumoniae* (in the absence of other otopathogens) [21,22]. However, it should be noted that these studies did not involve replication in a nasopharyngeal epithelial environment and the results were not compared with non-pathogenic bacteria [6].

To date, *A. otitidis* has been associated almost exclusively with MEF as a putative pathogen. There are few reports of *A. otitidis* as a causative agent of unrelated diseases. For example, *A. otitidis* has been described as the causative agent of endophthalmitis following intravitreal injection in two clinical cases [23], and endocarditis [24] developed as a complication of chronic OM. However, in both cases, the source of infection remained unclear. Recently, *A. otitidis* was isolated from a blood culture of a septic patient who had recently suffered an ear infection [25].

Because *A. otitidis* is a potential pathogen of the paranasal sinuses, the advent of molecular methods has significantly changed our understanding of the paranasal sinus microbiota. The culture-independent approaches have clearly demonstrated that healthy paranasal sinuses are not sterile and furthermore harbor complex polymicrobial communities [5,26,27]. Abreu et al. showed that the health of the paranasal sinus mucosa is highly dependent on the composition of its resident microbiota [26]. In a 2016 systematic literature review, Lee et al. reported greater species diversity in paranasal sinus aspirates from healthy individuals compared with diseased individuals and selective abundance of certain microbes in patients [27]. Reduced bacterial diversity and dysbiosis are now considered critical for disease occurrence. However, *A. otitidis* has not yet been described as part of the normal microbiota of the paranasal sinuses. In addition, common respiratory pathogens (*S. pneumoniae*, *H. influenzae*, *M. catarrhalis*, and *S. pyogenes*) and anaerobic bacteria are mentioned as causative agents in all the literature reviewed on acute sinusitis [3]. Sinusitis can be caused by multiple pathogens; polymicrobial infection is described in about one-third of reported cases. Sometimes the pathogen could not be isolated and grown in cultures at all. Brook found that pathogens could be isolated from sinus aspirates in only two-thirds of patients with acute sinusitis [3].

We now know that laboratory detection of organisms associated with sinusitis and OM can be unreliable if only culture-based methods are used. However, not all diagnostic laboratories have the equipment and expertise needed for non-culture-based methods.

Isolation of *A. otitidis* using standard culture methods is difficult because its growth rate is slow. Therefore, its small colonies on blood agar plates may be overlooked or mistaken for slow-growing viridans group streptococci. Since the initial growth of isolates often starts after 72 h, it would be better to extend the aerobic incubation to 3–4 days for middle ear/sinus samples. In addition to direct cultures, the use of enrichment culture media, such as brain heart infusion (BHI) broth, can improve cultivation efficiency [28]. The aerobic growth characteristics of *A. otitidis* may help distinguish the genus from other facultatively anaerobic Gram-positive cocci.

Published data suggest that a combination of culture-based and culture-independent methods can improve the detection rate of *A. otitidis*. Culture-independent methods can be used selectively to detect this largely overlooked organism in culture-sterile clinical specimens. There are no guidelines for antimicrobial susceptibility testing for this species. Our sensitivity results are consistent with those of Ashhurst-Smith et al. Our isolate was sensitive to beta-lactams, tetracycline, vancomycin, and co-trimoxazole [28].

**Table 1:** Review of literature on detection of *A. otitidis* from nasopharyngeal specimens.

Specimen	Diagnosis	Culture *n* (%)	PCR *n* (%)	Reference
SA	chronic sinusitis	0/27	2/27 (7)	[5]
NPS	OME	0/56	6/56 (11)	[11]
NPS	upper RTI	0/129	9/129 (7)	[29]
NPS	healthy children	NP	2/386 (0.5)	[30]
NPS	OME	0/34	0/34	[31]
NPS	healthy children	NP	4/50 (8)	[32]
NPS	healthy young adults	0/10	0/70	[33]

SA, sinus aspirate; NPS, nasopharyngeal swab; RTI, respiratory tract infection; OME, otitis media with effusion; NP, not performed.

Although the isolate described here was sensitive to amoxicillin, the drug of choice for acute sinusitis, the observed macrolide and clindamycin resistance may complicate treatment of patients with penicillin allergy.

Our literature search revealed no other reports of *A. otitidis* associated with acute sinusitis. This case report is unique because it reports the growth of the organism in pure culture on a conventional culture medium from a sinus aspirate. We believe that the detection of *A. otitidis* in sinus aspirate in pure culture may have clinical significance. This finding allowed us to assume that no other interfering bacteria were present and that *A. otitidis* indeed has its own pathogenicity. Further studies are needed to better define its role in the pathogenesis of sinusitis.

## Data Availability

Data supporting this case report are available from the corresponding author on reasonable request.

## References

[1] Dimitri-Pinheiro S., Soares R., Barata P. (2020). The Microbiome of the Nose—Friend or Foe?. Allergy. Rhinol..

[2] Arora H.S. (2018). Sinusitis in Children. Pediatric Ann..

[3] Brook I. (2011). Microbiology of sinusitis. Proc. Am. Thorac. Soc..

[4] Faden H., Dryja D. (1989). Recovery of a unique bacterial organism in human middle ear fluid and its possible role in chronic otitis media. J. Clin. Microbiol..

[5] Kalcioglu M.T., Durmaz B., Aktas E., Ozturan O., Durmaz R. (2003). Bacteriology of chronic maxillary sinusitis and normal maxillary sinuses: Using culture and multiplex polymerase chain reaction. Am. J. Rhinol..

[6] Lappan R., Jamieson S.E., Peacock C.S. (2020). Reviewing the Pathogenic Potential of the Otitis Associated Bacteria Alloiococcus otitidis and Turicella otitidis. Front. Cell. Infect. Microbiol..

[7] Güvenç M.G., Midilli K., Inci E., Kuşkucu M., Tahamiler R., Ozergil E., Ergin S., Ada M., Altaş K. (2010). Lack of Chlamydophila pneumoniae and predominance of Alloiococcus otitidis in middle ear fluids of children with otitis media with effusion. Auris Nasus Larynx.

[8] Allen E.K., Koeppel A.F., Hendley J.O., Turner S.D., Winther B., Sale M.M. (2014). Characterization of the nasopharyngeal microbiota in health and during rhinovirus challenge. Microbiome.

[9] (2020). EUCAST v. 10.0, The European Committee on Antimicrobial Susceptibility Testing. Breakpoint Tables for Interpretation of MICs and Zone Diameters, Version 10.0. http://www.eucast.org.

[10] Leskinen K., Hendolin P., Virolainen-Julkunen A., Ylikoski J., Jero J. (2002). The clinical role of Alloiococcus otitidis in otitis media with effusion. Int. J. Pediatric Otorhinolaryngol..

[11] Harimaya A., Takada R., Somekawa Y., Fujii N., Himi T. (2006). High frequency of Alloiococcus otitidis in the nasopharynx and in the middle ear cavity of otitis-prone children. Int. J. Pediatric Otorhinolaryngol..

[12] Farajzadah Sheikh A., Saki N., Roointan M., Ranjbar R., Yadyad M.J., Kaydani A., Aslani S., Babaei M., Goodarzi H. (2015). Identification of Alloiococcus otitidis, Streptococcus pneumoniae, Moraxella catarrhalis and Haemophilus influenzae in Children with Otitis Media with Effusion. Jundishapur. J. Microbiol..

[13] Folino F., Fattizzo M., Ruggiero L., Oriano M., Aliberti S., Blasi F., Gaffuri M., Marchisio P., Torretta S. (2021). Nasopharyngeal Microbiota Analysis in Healthy and Otitis-prone Children: Focus on History of Spontaneous Tympanic Membrane Perforation. Pediatric Infect. Dis. J..

[14] Sjövall A., Aho V.T.E., Hyyrynen T., Kinnari T.J., Auvinen P., Silvola J., Aarnisalo A., Laulajainen-Hongisto A. (2021). Microbiome of the Healthy External Auditory Canal. Otol. Neurotol..

[15] Chan C.L., Richter K., Wormald P.J., Psaltis A.J., Vreugde S. (2017). Alloiococcus otitidis Forms Multispecies Biofilm with Hamophilus influenzae: Effects on Antibiotic Susceptibility and Growth in Adverse Conditions. Front. Cell. Infect. Microbiol..

[16] Stewart P.S., Costerton J.W. (2001). Antibiotic resistance of bacteria in biofilms. Lancet.

[17] Donlan R.M., Costerton J.W. (2002). Biofilms: Survival mechanisms of clinically relevant microorganisms. Clin. Microbiol. Rev..

[18] Fergie N., Bayston R., Pearson J.P., Birchall J.P. (2004). Is otitis media with effusion a biofilm infection?. Clin. Otolaryngol. Allied. Sci..

[19] Himi T., Kita H., Mitsuzawa H., Harimaya A., Tarkkanen J., Hendolin P., Ylikoski J., Fujii N. (2000). Effect of Alloiococcus otitidis and three pathogens of otitis media in production of interleukin-12 by human monocyte cell line. FEMS Immunol. Med. Microbiol..

[20] Harimaya A., Koizumi J.-I., Fujii N., Himi T. (2007). Interleukin-8 induction via NF-κB, p38 mitogen-activated protein kinase and extracellular signal-regulated kinase1/2 pathways in human peripheral blood mononuclear cells by Alloiococcus otitidis. Int. J. Pediatric Otorhinolaryngol..

[21] Ashhurst-Smith C., Hall S.T., Burns C.J., Stuart J., Blackwell C.C. (2014). In vitro inflammatory responses elicited by isolates of Alloiococcus otitidis obtained from children with otitis media with effusion. Innate Immun..

[22] Harimaya A., Fujii N., Himi T. (2009). Preliminary study of proinflammatory cytokines and chemokines in the middle ear of acute otitis media due to Alloiococcus otitidis. Int. J. Pediatr. Otorhinolaryngol..

[23] Marchino T., Vela J.I., Bassaganyas F., Sánchez S., Buil J.A. (2013). Acute-Onset Endophthalmitis Caused by Alloiococcus otitidis following a Dexamethasone Intravitreal Implant. Case Rep. Ophthalmol..

[24] Guler A., Sahin M.A., Gurkan Yesil F., Yildizoglu U., Demirkol S., Arslan M. (2015). Chronic Otitis Media Resulting in Aortic Valve Replacement: A Case Report. J. Tehran Heart Cent..

[25] Yaghoubian J.M., Tirado A., Falkowski R., Aleman V., Oyesanmi O., Tucci V. (2020). Alloiococcus otitis—An Unusual Cause for Bacteremia: A Case Report. Acta Sci. Otolaryngol..

[26] Abreu N.A., Nagalingam N.A., Song Y., Roediger F.C., Pletcher S.D., Goldberg A.N., Lynch S.V. (2012). Sinus microbiome diversity depletion and Corynebacterium tuberculostearicum enrichment mediates rhinosinusitis. Sci. Transl. Med..

[27] Lee J.T., Frank D.N., Ramakrishnan V. (2016). Microbiome of the paranasal sinuses: Update and literature review. Am. J. Rhinol. Allergy.

[28] Ashhurst-Smith C., Hall S.T., Walker P., Stuart J., Hansbro P.M., Blackwell C.C. (2007). Isolation of Alloiococcus otitidis from Indigenous and non-Indigenous Australian children with chronic otitis media with effusion. FEMS Immunol. Med. Microbiol..

[29] Tano K., von Essen R., Eriksson P.O., Sjöstedt A. (2008). Alloiococcus otitidis--otitis media pathogen or normal bacterial flora?. AMPIS.

[30] Janapatla R.P., Chang H.J., Hsu M.H., Hsieh Y.C., Lin T.Y., Chiu C.H. (2011). Nasopharyngeal carriage of Streptococcus pneumoniae, Haemophilus influenzae, Moraxella catarrhalis, and Alloiococcus otitidis in young children in the era of pneumococcal immunization, Taiwan. Scand. J. Infect. Dis..

[31] Aydin E., Taştan E., Yücel M., Aydoğan F., Karakoç E., Arslan N., Kantekin Y., Demirci M. (2012). Concurrent assay for four bacterial species including alloiococcus otitidis in middle ear, nasopharynx and tonsils of children with otitis media with effusion: A preliminary report. Clin. Exp. Otorhinolaryngol..

[32] Durmaz R., Ozerol I.H., Kalcioglu M.T., Oncel S., Otlu B., Direkel S., Hendolin P.H. (2002). Detection of Alloiococcus otitidis in the nasopharynx and in the outer ear canal. New Microbiol..

[33] De Baere T., Vaneechoutte M., Deschaght P., Huyghe J., Dhooge I. (2010). The prevalence of middle ear pathogens in the outer ear canal and the nasopharyngeal cavity of healthy young adults. Clin. Microbiol. Infect..

